# Preparing Root Canals for Filling

**Published:** 1904-06

**Authors:** J. W. Daniels

**Affiliations:** Monon, Ind.


### PREPARING ROOT CANALS FOR FILLING.

By J. W. Daniels, Monon, Ind.

The root canals are cleansed and dried in the usual way. Before filling with chloro-percha and gutta-percha points, tinc. aconite is pumped into the canals by means of a broach wrapped with cotton, after which the surplus aconite is removed from the pulp chamber with a pellet of cotton. The canals can now be filled with chloro-percha and gutta-percha cones in the ordinary way and no soreness will result. The action of the tinc. aconite on the tissue in the apical region prevents inflammation. I have not had an instance of patients complaining of soreness of the tooth since I adopted this method.—*Review.*

